# Highly Adaptable and Biocompatible Octopus‐Like Adhesive Patches with Meniscus‐Controlled Unfoldable 3D Microtips for Underwater Surface and Hairy Skin

**DOI:** 10.1002/advs.201800100

**Published:** 2018-04-30

**Authors:** Sangyul Baik, Jiwon Kim, Heon Joon Lee, Tae Hoon Lee, Changhyun Pang

**Affiliations:** ^1^ School of Chemical Engineering Sungkyunkwan University (SKKU) Seobu‐ro, Jangan‐gu Suwon Gyeonggi‐do 440‐746 Republic of Korea; ^2^ Department of Electrical Engineering Kwangwoon University Seoul 139‐701 Republic of Korea; ^3^ SKKU Advanced Institute of Nanotechnology (SAINT) Samsung Advanced Institute for Health Science & Technology (SAIHST) Sungkyunkwan University (SKKU) Seobu‐ro, Jangan‐gu Suwon Gyeonggi‐do 440‐746 Republic of Korea

**Keywords:** biomimetics, dry adhesive, meniscus, nanostructures, suction cups

## Abstract

Adhesion capabilities of various skin architectures found in nature can generate remarkable physical interactions with their engaged surfaces. Among them, octopus suckers have unique hierarchical structures for reversible adhesion in dry and wet conditions. Here, highly adaptable, biocompatible, and repeatable adhesive patches with unfoldable, 3D microtips in micropillars inspired by the rim and infundibulum of octopus suction cup are presented. The bioinspired synthetic adhesives are fabricated by controlling the meniscus of a liquid precursor in a simple molding process without any hierarchical assemblies or additional surface treatments. Experimental and theoretical studies are investigated upon to increase the effective contact area between unfoldable microtips of devices, and enhance adhesion performances and adaptability on a Si wafer in both dry and underwater conditions (max. 11 N cm^−2^ in pull‐off strength) as well as on a moist pigskin (max. 14.6 mJ peeling energy). Moreover, the geometry‐controlled microsuckers exhibit high‐repeatability (over 100 cycles) in a pull‐off direction. The adhesive demonstrates stable attachments on a moist, hairy, and rough skin, without any observable chemical residues.

Biologically inspired adhesion strategies based on nanoscale hierarchical structures without any chemical moieties are known to induce mechanical interlocking or molecular interactions.^1^, ^2^, ^3^ These architectures can be applied to develop reversible and repeatable skin adhesive patches for wound protection and smart skin‐attachable devices.^4^, ^5^ Conventional skin‐adhesives are usually acrylic‐based, thus repeatable and residue‐free adhesion against moist and nonflat skins cannot be expected.^6^ In fact, the detachment of conventional glue‐based adhesives around moist wound areas may cause skin damage and contamination. Hence, reversible, biocompatible, and contamination‐free bandages or pads are highly needed for transdermal patches with therapeutic systems or skin/organ‐attachable devices with diagnostic sensing elements for long‐term, vital signal monitoring.^7^

To date, novel materials such as gecko‐inspired dry adhesives with hierarchical architectures,^4^, ^6^, ^8^, ^9^, ^10^, ^11^ microneedle‐based patches,^2^ and chitosan‐based adhesives with dissipative matrices,^12^ have been reported. Despite impressive performances of these adhesives, developing a new type of skin patch capable of conformal attachment, high repeatability, and contamination‐free, remains a challenge. This is because skin is known to have extraordinary stretchability (ε > 100% where ε is the strain),^13^ high roughness (maximum height ≈40 µm and root‐mean‐square (RMS) ≈22 µm),^14^ and is often covered with moisture and numerous hairs.

Recently, suction‐cups of octopi have received much attention due to strong, reversible adhesion performances against dry, wet, and irregularly rough surfaces.^15^, ^16^ Inspired by the architecture of octopi suction cups, various artificial adhesives with miniaturized suckers have been developed. For example, artificial microsuckers (μ‐SCs) showed thermal‐responsive reversibility on dry Si‐wafer,^10^ or were integrated with medical sensors.^1^ However, their engagements were limited to dry surfaces, while adhesion capability against wet surfaces and rough skin need to also be demonstrated for proper skin applications. Specifically, the dome‐like protuberances in octopus‐suckers were investigated to develop a reversible and clean adhesive under various wet conditions.^11^ The adhesive showed high adhesion performances in the normal direction due to strengthened suction effect assisted by capillarity, but was based on cavities of a flat, elastic substrate without any extruded structures. Therefore, its adaptability and peeling strength against wet, hairy, and nonflat skin need to be improved to widen its versatility. Micro/nanopillars with concave shapes at the tip, inspired by the extruded portion of octopus suckers,^1^, ^8^, ^17^, ^18^, ^19^ have been developed for advantageous contacting and conformal sealing against rough substrates.^20^ The microsuckers,^18^, ^19^, ^21^ however, are not applicable to skin‐patches due to their thick and rigid backbone substrates. Moreover, the suction‐cups at the tip are relatively small and generate insufficient adhesion‐strength under water conditions, due to high curvature radii of microsuckers. In case of nanosucker array showing high adhesion forces in dry/wet conditions,^8^ the polymeric nanostructures could be easily deformed after repetitive attachments and detachments. Additionally, adhesion performances against wet surface and microtopographic skin were not demonstrated. Fabricating miniaturized octopus‐inspired adhesives require costly, microscale molds, based on multiple steps of photolithography and chemical synthesis, which limit the curvatures of the artificial suction cups. Therefore, simple solution‐based methods to improve adhesion on dry, wet, and even hairy skin need to be developed for versatile applications of reversible glue‐free skin‐adhesives.

In this study, we developed biocompatible, highly reversible skin‐adhesive patches with arrays of unfoldable, meniscus‐controlled, 3D microtips, fabricated through a simple polymeric replication method. In contrary to the previous study mimicking dome‐like protuberances located at lower chambers in octopus suction cups,^11^ this work aims to develop the novel dry adhesive based on the adhesion mechanism of the circumferential rim and infundibulum located at their upper chambers. The bioinspired protruding structure can be effective in adaptability as well as stability against rough surfaces, enhancing dry/wet adhesion strength in both pulling and peeling directions. The devices showed strong adhesion in normal and peeling directions on dry and wet surfaces, as well as on hairy skin with dual‐roughness morphology. Various μ‐SCs were designed to maximize the contact area of the suction stress by controlling the shapes in μ‐SCs tips using different microhole‐patterned molds. Specifically, the heights of the μ‐SCs were orchestrated with the interfacial tension of the precursor and an externally applied pressure. Due to different surface energies of hole‐patterned molds, the modulated menisci of the prepolymer (soft‐poly(urethane acrylate); s‐PUA) showed curvatures varying from 0.66 to 0.93, which originate from simple wetting and capillary behaviors. With interfacial energy balance, we investigated simple methodological models to maximize the volume of suction chambers in octopus‐like architectures and enhance adhesion forces and adaptability with unfoldable 3D microtips on highly rough surfaces covered with hair. Using a soft polyurethane‐based elastomer to produce artificial microsuckers (diameters of 100 µm), we noticed that the device with the suction‐cups of 0.93 curvature exhibited the strongest reversible adhesion strength in a pull‐off direction against a wafer in dry (≈3 N cm^−2^) and underwater (≈11 N cm^−2^) conditions with high repeatability (over 100 cycles), as well as peel‐off direction (dry: ≈0.5 N, underwater: ≈1.0 N). The patches with μ‐SCs, composed of a biocompatible, softened polydimethylsiloxane, stably attached to wet pig skin, on which the measured forces were ≈1.8 N cm^−2^ in normal direction and ≈13.5 mJ in peeling energy.

In the attachment strategy of octopus suckers, based on muscular hydrostats, the soft, infundibular portion is critical for its ability to comply with the engaged rough surface and prevent leakage of water molecules into the orifice of suckers.^16^, ^20^, ^22^ We made close observations of suction‐cups of the *Octopus vulgaris* (the most common species of octopus), shown in the inset image of **Figure** **1**a, to notice a disk‐like acetabular roof having grooved surfaces encircled by an extruded rim of epithelium around the edge of the sucker. The columnar rim, in particular, plays a critical role for high adaptability on various surface morphologies in wet conditions to completely lock the suction chamber,^18^, ^23^ while the wrinkled surface in the acetabular roof maximizes the contact area during attachment.^18^, ^23^ To mimic the structure of the circumferential rim and acetabular roof in octopus suction cups, we controlled the meniscus of a liquid precursor (s‐PUA) by trapping air during replication, as shown in Figure 1b. At stage I of Figure 1c(i), the liquid precursor with a 3D curvature is partially filled in a hole‐patterned mold (diameter 100 µm; height 100 µm) of hexagonal array with SR 1 (spacing ratio; the distance between each structure (*d*) divided by the diameter (2*r*)) due to the capillary from the interfacial energy balance. After gently placing a transparent film (PET) on the coated precursor layer, we applied a uniform, external load to maximize the height of fibers with suction cups (see Stage II of Figure 1c(ii); and Figure S1, Supporting Information). To enhance the 3D curvature in the solid mold by capillary‐action, various molds made of s‐PUA, (tridecafluoro‐1,1,2,2‐tetrahydrooctyl)‐trichlorosilane (FOTCS)‐treated silicon, and polydimethylsiloxane (PDMS) were used as shown in Figure S2 (see the Experimental Section for details, Supporting Information). After curing the meniscus‐controlled precursor through UV exposure, uniform arrays of μ‐SCs, inspired by the infundibulum and rim of octopi suckers, can be obtained as shown in Figure 1d–f.

**Figure 1:**
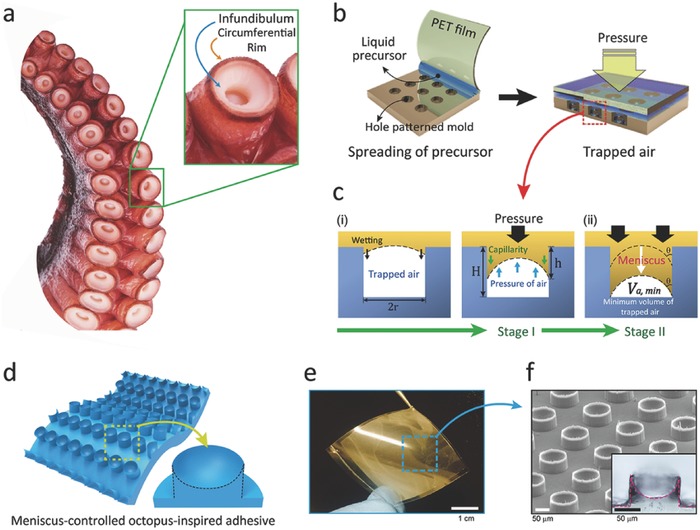
a) Image of an octopus sucker (*Octopus vulgaris*) with infundibular and circumferential rim. b) Solution processing of μ‐SC array by controlling capillarity and external pressure. c) Detailed mechanisms underlying controlled meniscus (stage I) and external pressure (stage II). d,e) Illustrations d) and photograph e) of octopus‐inspired adhesive patches with meniscus‐controlled, unfoldable 3D microtips (≈3 cm × 3 cm). f) SEM image and cross‐sectional optical image of μ‐SCs (100 µm diameter and 75 µm height) inspired by the infundibular and circumferential rim of an octopus sucker.

A simple model was designed to explain the controllable capillarity behavior in the fabrication of μ‐SCs. For suction chambers in μ‐SCs, the size of trapped air‐bubbles are determined by an energy balance among the pressure of trapped air (*P*_a_; blue arrow), Laplace pressure by capillarity^24^ (*P*_L_; green arrow), and the external pressure induced by a compressor (*P*_C_; black arrow), as illustrated in Figure 1c: *P*_a_ = *P*_L_ + *P*_c_. Here, the Laplace pressure by capillarity (*P*_L_) in trapped air is given by *P*_L_ = (2γcosθ/*r*), where *r* is the radius of the hole‐pattern, γ is the interfacial tension, and θ is the contact angle between a liquid precursor and the side wall of a cylindrical hole in the solid mold (see Figure 1c(ii); and Figure S3, Supporting Information). In compliance with Boyle's law, the 3D curvature and the meniscus‐height (*h*) are controlled by uniformly applying pressure *P*_c_ with a compressor through the following equation (see the Supporting Information for details)

(1) $$h\;=\left(1-\frac{P_{0}}{P_{L}+P_{C}}\right)\;H+r\;\tan\theta \left(\sec\theta -\frac{1}{3}\tan\theta \right)$$

where, *P*_0_ is the initial air‐pressure in ambient condition (≈101 kPa), *H* is the height of the hole‐patterned mold (≈100 µm), and *r* is the radius of the cylindrical hole (≈50 µm). Specifically, the geometry of concave suction chambers in micropillars follows the shape of the trapped air bubble in the mold, which is determined by θ due to tiny shrinkage (<0.7%) via polymerization of s‐PUA after UV‐curing.^25^ The height of μ‐SCs was observed as ≈75 µm after applying a gentle load (300 kPa) with a compressor (*P*_C_) (see the Supporting Information for detailed methods). For structural uniformity, we applied a uniform load to a transparent cover‐film (PET) on the precursor‐coated mold, after the viscous solution reached equilibrium state of capillary action without external pressure (see Figure 1c). Subsequently, the liquid precursor with preciously controlled air bubbles was exposed to UV light from the light source located in the loading plate of the compressor (see Figure S1, Supporting Information) to obtain the array of meniscus‐controlled octopus‐like microfibers. With this, we fabricated the octopus‐inspired adhesive (3 × 3 cm^2^) with highly concave microtips composed of a polymeric mold (s‐PUA), as shown in Figure 1d. We verified the architecture with a tilted scanning electron microscope (SEM) image and inset cross‐sectional optical image in Figure 1e,f.

The 3D concave microtips were controlled using solid molds with varying surface energies: FOTCS‐treated silicon (≈60 mJ m^−2^), s‐PUA (≈40 mJ m^−2^), and PDMS (≈20 mJ m^−2^).^6^, ^26^ As shown in **Figure** **2**a, trapped air bubbles are located at the bottom surfaces of different hole patterns due to energy minimization after controlling the menisci, with measured contact angle (θ) ranging from 10^o^ to 70° for the three solid molds. Meanwhile, the array of flat, cylindrical pillars was obtained after removing air bubbles in Figure 2a. In Figure 2b, curvature‐ratios (*CR*) between *r* and *R* (the radius of curvature) are plotted against different contact angles. This result is in agreement with our prediction, *CR = r/R =* cosθ (see Figure 2b, see the Supporting Information for details). Based on our observations (see Figure 2c–f), the array of μ‐SCs fabricated with the s‐PUA‐based mold had the highest meniscus curvature because of greater molecular interactions than other molds.^27^ By controlling the meniscus of a viscous precursor, we obtained adhesive patches with uniform, fiber arrays of different 3D microtips, tailored by capillarity as shown in Figure 2c–f; and Figure S4, Supporting Information.

**Figure 2:**
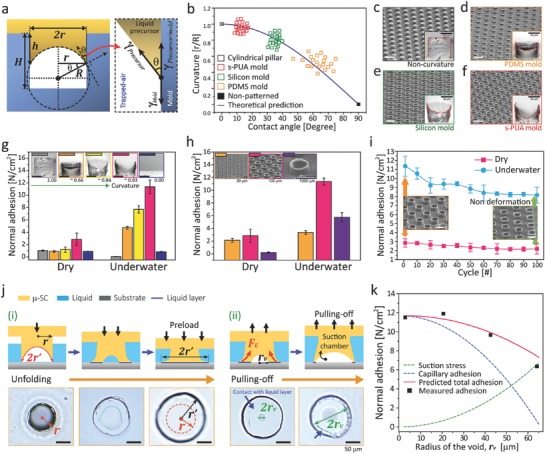
a) Schematics showing the curvature of unfoldable 3D microtips controlled by molecular interactions between the precursor and the solid‐mold. b) Predicted and experimental curvatures (*r/R)* of various solid molds. c–f) Tilted SEM images and cross‐sectional OM images of various architectures: c) cylindrical pillars without trapped airs, μ‐SCs fabricated by d) PDMS mold, e) FOTCS‐treated silicon mold, f) s‐PUA mold. Here, scale bars (white) correspond to 200 µm. g) Dry/wet adhesion strengths for various μ‐SC arrays with 5 different microtips. h,i) Dry/wet adhesion performances for 3 μ‐SCs in different sizes (15, 50, and 500 µm in diameter) with ≈0.93 curvature. j) Detailed unfolding (i) and pulling‐off (ii) processes of a 3D microtip in a microsucker and corresponding images in wet surface. k) Wet‐adhesion profiles for predictions (suction stress, capillary interaction, and total adhesion) and measured data with respect to radius of a void (*r_v_*) between a 3D microtip and a substrate as the adhesive is pulled off. Error bars represent standard deviations (for g–i; *N* = 10).

In Figure 2g, we investigated the adhesion performances of the patches with different curvatures at the tips (see images of Figure 2c–f). Normal adhesion forces on a Si wafer in dry and wet conditions were measured using a custom‐built equipment at room temperature (see Figure S5a,b, Supporting Information). Here, the engaged surfaces of dry (left) and underwater (right) conditions are displayed in the inset photographs of Figure S5b (Supporting Information). Based on this method, Figure 2g presents the collective data of adhesion strengths created by various μ‐SC‐arrays with five different structural features for the s‐PUA‐based microstructures (diameter of 100 µm) as shown in the inset images of Figure 2g: three different concave pillars, cylindrical pillar without curvature, and a flat sample. In Figure 2g, pull‐off adhesion forces measured on a dry surface were compared to those against an underwater surface. In our measurements, the artificial adhesive produced by the s‐PUA mold (*CR* ≈ 0.93) exhibited the highest adhesion strength for both dry (≈3 N cm^−2^) and underwater (≈11 N cm^−2^) conditions. In Figure 2g, the pull‐off adhesion forces in both conditions increases along with the curvature of suction‐chambers in pillar array (*CR*: 0.66–0.93). This behavior is mainly attributed to the enlarged 3D microtips of octopus‐like sucker that can increase effective contact area to a combining surface. Therefore, the suction stress (negative pressure effect for wet condition) and molecular interactions (van der Waals for dry surface or capillary interaction for wet condition) between two engaged surfaces are amplified.^6^, ^8^, ^28^ Likewise, the adhesive with *CR* of 0.93 exhibited the highest adhesion performances among other samples for peeling off in both dry and underwater conditions (see Figure S6 for details, Supporting Information). The detailed mechanisms underlying wet adhesion will be discussed later. To understand the size effect of meniscus‐controlled μ‐SCs, we measured the pull‐off adhesion strengths of various microsucker arrays with three different diameters fabricated using an s‐PUA mold (30, 100, or 1000 µm; see insets of Figure 2h) under the same preload of 3.5 N cm^−2^. In Figure 2h, the array of microsuckers with 100 µm in diameter shows higher performances than other arrays against dry and wet surfaces, because the volume of suction‐chambers was maximized with densely populated with μ‐SCs (≈ 5 × 10^3^ #cm^−2^). Notably, the adhesive with hexagonally arranged, size‐controlled μ‐SCs (diameter of 100 µm) showed high repeatability (over 100 cycles) against both dry and wet surfaces without dramatic degradations (see Figure 2i). No structural collapses occurred after multiple attachments and detachments, as shown in inset SEM images of Figure 2i.

To explain the enhancement of adhesion strength of the tailored sucker array in wet conditions, we propose a simple methodological model underlying the suction effect and capillarity‐interaction between unfoldable 3D microtips and an engaged surface. The maximum adhesion force, in particular, can be explained with the collective of suction stress (σ_s_) and capillary interaction (σ_c_) induced by a single μ‐SC: σ_total_ = σ_s_  + σ_c_. As a preload (<3.5 N cm^−2^) is applied to the artificial adhesive, the tailored 3D microtips unfold to increase the effective contact area on a surface as shown in Figure 2j. During attachment, the inside of suction chambers can enlarge to reach vacuum state, which results from the deformation of elastic pillars by an applied preload (see Stage I and OM images in Figure 2j; and Figure S7, Supporting Information). The adhesion via capillary‐bridges (σ_c_) between engaged surfaces is maximized, which we express with the radius of the unfolded tip (*r*′) (see images of Figure 2j(i)). Continuously, the suction effect (σ_s_) takes place as the μ‐SCs are pulled off (see images of Figure 2j(ii) and Figure S7, Supporting Information), causing void formation inside the structures. Herein, the size of the void can be estimated as *r*_v_. Based on our assumption, total adhesion stress of the tailored suckers can be obtained with dynamic contact area $\left(A_{\mathrm{dynamic}}=\;\pi \left(r\prime^{2}-r_{v}^{2}\right)\right)$ of enlarged tips induced by the competition between the suction effect and the capillarity (see Figure S8b for details, Supporting Information)

(2) $$\;\sigma_{\mathrm{total}}=\Delta P_{\max}\;\pi r_{v}^{2}n+\pi (r\prime^{2}-r_{v}^{2})\gamma n\;\left(\frac{\cos\theta_{1}+\cos\theta_{2}}{h\prime }+\frac{2}{r\prime -r_{v}}\right)$$

Here, Δ*P*_max_ is the pressure difference between the ambient pressure and the pressure inside the μ‐SC (nearly creating a vacuum state; ≈101 kPa), *n* is the number of μ‐SCs per unit area (≈ 5  × 10^3^ # cm^−2^), γ is the surface tension of the water (≈0.072 J m^−2^), θ_1_ is the contact angle of D.I. water on the substrate (silicon wafer), θ_2_ is the contact angle of D.I. water on the μ‐SC surface (s‐PUA; see Figure S9, Supporting Information), and *h′* is the height of the liquid film (≈0.25 µm). To prevent the interference among μ‐SCs during their unfolding behaviors, we designed our adhesive patches (*r*′ − *r* < *d*; see Figures S7–S8, Supporting Information) so that the μ‐SCs cannot physically contact each other in all experiments when completely unfolded (Figure S7b, Supporting Information). By monitoring the void radius (*r*_v_), we could predict the adhesive's suction stress, capillarity adhesion, and total adhesion strength (red lines) as shown in Figure 2k; this is well in agreement with our experimental data (black dots).

For practical applications to versatile skin‐adhesives, we fabricated μ‐SCs using modulus‐tuned PDMS (elastic modulus ≈0.8 MPa) as shown in **Figure** **3**a,b. An s‐PUA‐based mold was used to obtain the thin PDMS‐based patch with μ‐SCs (3 × 3 cm^2^, *CR* ≈0.93, and thickness ≈600 µm; see the Experimental Section and Figure S10, Supporting Information).^9^ As shown in Figure 3d–g, the patch's adhesion performances in pull‐off and peeling directions were measured against a postmortem pigskin (3 × 2 cm^2^) with dual‐roughness and undulations at a wide range of scales from a few micrometers to a few milimeters (see Figure 3c; see the Supporting Information for detailed measurements). From obervations, the microscale roughness of the postmortem pigskin (RMS) was ≈48.5 µm, which was calculated using a morphological profile (see yellow line in Figure 3c(i), (ii) for surface profile, and (iii) for cross‐sectional SEM image). The patch's nanoscale roughness, on the other hand, was measured at ≈202 nm through atomic force microscopy (see Figure S11, Supporting Information). The pull‐off adhesion forces of the PDMS adhesive with μ‐SCs were relatively high on pigskin in dry, moist conditions (≈1.5 N cm^−2^ and ≈1.8 N cm^−2^, respectively) compared to those of a soft, flat PDMS sample (see Figure 3d; and Figure S12, Supporting Information and for detailed method). Here, the moist pigskin was covered with droplets (40% of areal fraction). With the octopus‐like suckers, the patch had a dramatic increase in pulling strength for dry/moist condtions due to enhanced suction stress and amplified molecular interactions in the unfoldable 3D microtips (see Figure 3d). In Figure 3e–g, the device on dry skin shows moderately high peeling energy (Max. *W*_ad, P_ ≈14.6 mJ), which could be estimated with a time‐dependent peeling curve (see yellow area in Figure 3e). The octopus‐inspired architecture with enlarged tips could maintain its adhesive properties during initial propagation of the peeling process, allowing a more stable attachment to the engaged pigskin (see Figure 3f,g). Moreover, to demonstrate versatility as a stable skin adhesive, we measured the patch's adhesion performances on a moist and rough pigskin with or without relatively large hairs (Figure S13, Supporting Information). All animal experiments were carried out in accordance with Institutional Animal Care and Use Committee (IACAC) of Sungkyunkwan University approval (SKKUIACUC‐2016‐10‐0001‐2; see Supporting Information for details). Shown in Figure S13e,f (Supporting Information), the octopus‐inspired adhesive patch exhibits higher pull‐off and peel‐off strengths than those on a flat sample on hairy pigskin in dry, moist, and underwater conditions. Noteably, the photographic images in Figure 3h,i indicate how the μ‐SC‐populated patch (2 × 2 cm^−2^) can withstand a total weight of 0.5 kg attached on a rough skin covered with hairs. Hardly any residues such as chemical contaminations, traces of dust particles, and other impurities, were noticeable on our volunteer's hands, on which we attached our patch for nearly 6 h, after receiving Institutional Review Board (IRB) approval for both subject and parental assent (IRB approval number: SKKU 2016‐10‐010‐001) (see Supporting Information for details). We also witnessed that the adhesive showed greater peeling strength than the nonpatterned PDMS patch, as shown in Figure 3j.

**Figure 3:**
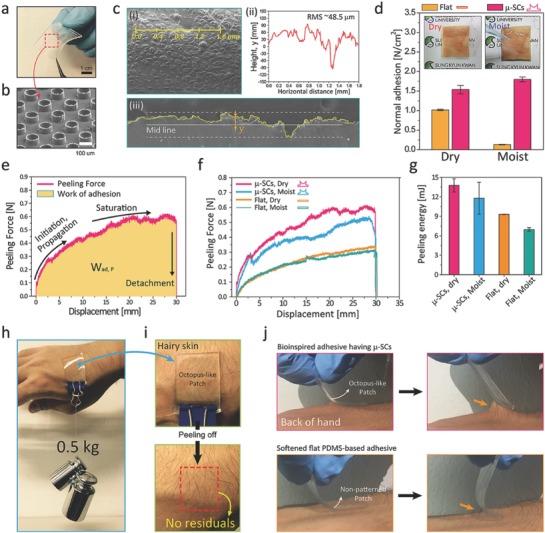
a,b) Photograph a) and SEM b) image of octopus‐like biocompatible PDMS‐based adhesive with enlargeable 3D microtips and μ‐SCs (100 µm in diameter and 75 in µm height: 3 cm × 3 cm and ≈600 µm thickness). c) Morphology analysis of an engaged pigskin with dual‐roughness (see yellow line): top‐view (i), surface‐profile (ii), Cross‐sectional SEM image of macroscale fluctuations (iii). d) Pull‐off strengths for a PDMS‐based μ‐SCs and a nonpatterned PDMS‐patch against a rough pigskin. Here, inset images show dry and moist pigskins. e) Representative displacement‐dependent profile of peeling adhesion on a pigskin. f,g) Peeling adhesion profiles f) and peeling energies g) for two different adhesives (PDMS‐based μ‐SCs and a nonpatterned patch) on dry and moist pigskin. h,i) Demonstration of the bioinspired adhesive (2 × 2 cm^2^) fully supporting a 0.5 kg weight on hairy skin h) without sticky residuals after detachment i). j) Photographs of an octopus‐inspired adhesive on the hairy skin of a volunteer's hand, indicating higher peel‐strength than a flat, PDMS‐based adhesive. Error bars represent standard deviations (for d and g; *N* = 10).

We propose octopus‐inspired adhesive patches with meniscus‐controlled unfoldable 3D microtips, fabricated using a simple, capillary‐assisted molding process. The unfolding concave geometry‐controlled microsucker tips showed high dry/wet adhesion performances in both pull‐off and peeling directions against a Si wafer and rough, hairy skin. These results imply that our adhesive patch design with enlargeable 3D microtips may pave the way for developing clean, conformal patches for wound‐healing and smart skin/organ‐attachable medical devices.

## Conflict of Interest

The authors declare no conflict of interest.

## Supporting information

Supplementary
