# Supplementary material for: Highly Adaptable and Biocompatible Octopus‐Like Adhesive Patches with Meniscus‐Controlled Unfoldable 3D Microtips for Underwater Surface and Hairy Skin
Source: Adv Sci (Weinh). 2018 Apr 30;5(8):1800100. doi: 10.1002/advs.201800100 (PMC6097001; doi:10.1002/advs.201800100)
Supplement: Supplementary file 1 — Supplementary [file ADVS-5-1800100-s001.pdf]

Copyright WILEY-VCH Verlag GmbH & Co. KGaA, 69469 Weinheim, Germany, 2018.

ADVANCED SCIENCE
Open Access

Supporting Information

for *Adv. Sci.,* DOI: 10.1002/advs.201800100

Highly Adaptable and Biocompatible Octopus-Like Adhesive Patches with Meniscus-Controlled Unfoldable 3D Microtips for Underwater Surface and Hairy Skin

*Sangyul Baik, Jiwon Kim, Heon Joon Lee, Tae Hoon Lee, and Changhyun Pang**

DOI: 10.1002/ advs.201800100
Article type: Communications

Highly Adaptable and Biocompatible Octopus-like Adhesive Patches with Meniscus-Controlled Unfoldable 3D Micro-Tips for Underwater Surface and Hairy Skin

*Sangyul Baik, Jiwon Kim, Heon Joon Lee, Tae Hoon Lee, and Changhyun Pang**

S. Baik, J. Kim, H. J. Lee, Prof. C. Pang
School of Chemical Engineering
Sungkyunkwan University (SKKU)
Seobu-ro, Jangan-gu, Suwon, Gyeonggi-do, 440-746, Republic of Korea

Prof. T.H. Lee
Department of Electrical Engineering, Kwangwoon University, Seoul 139-701, Korea

Prof. C. Pang
SKKU Advanced Institute of Nanotechnology (SAINT)
Samsung Advanced Institute for Health Science & Technology (SAIHST)
Sungkyunkwan University (SKKU)
Seobu-ro, Jangan-gu, Suwon, Gyeonggi-do, 440-746, Republic of Korea
E-mail: chpang@skku.edu

Keywords: Suction cup, Nanostructure, Meniscus, dry adhesive, biomimetics

Fabrication of octopus-inspired adhesives having μ-SCs. The hole-patterned molds made of silicon (30 μm in diameter, AR 1, and SR 1) were prepared by photolithography and subsequent reactive ion etching. Here, AR is the width-to-depth ratio and SR is the distance between each structure (*d*) divided by the diameter (*2r*). The molds were treated with a fluorinated self-assembled monolayer (SAM) solution ((tridecafluoro-1,1,2,2-tetrahydrooctyl)-trichlorosilane:FOTCS, Gelest Corp, Morrisville, PA, USA) diluted to 0.03 M anhydrous heptane (Samchun chemical, Pyeongtaek-si, Gyeonggi-do, Korea) in an Ar atmosphere. Using cyanoacrylate glue, the as-prepared molds were fixed to the upper side of a custom-built pressing machine as shown in Figure S1a. Afterwards, a liquid precursor (s-PUA:MINS-301RM purchased from Minuta Tech, Korea) was dispensed onto a polyethylene terephthalate film (PET; thickness 50 μm for a backbone) (see Figure S1a). The polymeric precursor and hole-patterned mold were then compressed with a pressure of 300 kPa to preciously control the capillarity of the precursor between a mold and a PET film as illustrated in Figure 1b-c. Subsequently, ultraviolet-light located in the pressing unit was irradiated for 2 minutes (see Figure S1b). By peeling the cured S-PUA/PET film off, we obtained octopus-inspired adhesives with μ-SCs. (see Figure S1c). The PUA replica was further exposed to ultraviolet light for several hours to achieve complete curing. To control the curvature of 3D micro-tips in the octopus-inspired adhesive (See Figure 2c-f and Figure S4), we used solid molds (s-PUA, silicon, and PDMS) of different surface energies (see Figure S3) to fabricate various μ-SCs in the same manner. The various solid molds obtained through multiple replications using Si-based molds are shown in Figure S2. For scale studies as shown in Figure 2h, we used s-PUA molds of 3 different diameters for the hole-patterns (30 μm, 100 μm, and 1000 μm with AR 1 and SR 1) and fabricated various μ-SCs through the same method. By contrast, the cylindrical micro-pillars were fabricated by removing air

bubbles completely by using a vacuum pump as shown in Fig. 2c. Details regarding the synthesis and characterization of the PUA polymers can be found elsewhere[1].

Fabrication of the softened PDMS-based skin-patches with and without μ-SCs. To obtain the skin-patch, we used soft, elastic PDMS (Polydimethylsiloxane: Sylgard, 184, Dow Corning), produced by mixing a curing agent (5 wt%) with a PDMS precursor. The soft PDMS-based patch with μ-SCs was fabricated through multiple replications using the as-prepared adhesive with size-maximized μ-SCs made using a s-PUA mold (*CR*~0.93) as shown in Figure S10. The mixture was then cured at 70 °C for 2 hours. On the other hand, PDMS-based patch without microstructures was fabricated with a flat FOTCS-treated Si-wafer. Herein, the thickness of the adhesives (~ 600 μm) was controlled by using a spin-coater.

Detailed mechanisms to control the geometry of μ-SC. To explain the underlying mechanism for tailoring the geometry of μ-SCs, a simple model based on the liquid-meniscus in a capillary action was investigated. As shown in Fig. 1c, the particular geometry of μ-SCs is achieved through stress balance between pressure of pressed air (P_a; blue arrow), Laplace pressure by capillarity [2] (P_L; green arrow), and the external pressure loaded by a compressor (P_C; black arrow): $P_a = P_L + P_c$. Here, the Laplace pressure (P_L) in trapped air is given by $P_L = (2\gamma cos\theta/r)$, where r is the radius of the hole-pattern of mold, γ is the interfacial tension, and θ is the contact angle between a liquid precursor and solid molds (see Figure 1c (i) and Figure S3). According to Young's equation[4], the 3D curvature of the meniscus of a polymer precursor results from the contact angle (θ) on the side-wall of a cylindrical-hole in a mold as shown in Figure 2a.

$$cos\theta = \frac{\gamma_{mold/trapped\ air} - \gamma_{precursor/mold}}{\gamma_{precursor/trapped\ air}} \quad (1)$$

We used 3 different patterned-molds of various surface energies: silicon (~60 mJ/m^2), s-PUA (~40 mJ/m^2), and PDMS (~20 mJ/m^2)[3]. For convenience, we defined the curvature-ratio (CR) of meniscus as $r/R = cos\theta$, resulting in the tip geometry of μ-SCs as shown in Figure 2b and d-f. Here, R is the radius of trapped-air.

In addition, the meniscus-height (h) in Figure 1c may determine the height of μ-SCs after curing process. Herein, the meniscus-height (h) can be controlled by external load of the pressing machine. Since the volume of a trapped air (V_a) is estimated with the energy balance between Laplace pressure (P_L) via capillarity an external load (P_C), we can obtain the following equation (2) by Boyle's law:

$$V_a = \frac{P_0 V_0}{P_L + P_C} \quad (2)$$

Here, P_0 is the initial air-pressure at ambient condition (~101 kPa) and V_0 is the volume of cylindrical hole ($\pi r^2 \cdot H$), wherein H is the depth of cylindrical hole. Because the curvature of the meniscus is decided by surface properties of solid molds (see Figure 1c (i)), the volume of trapped air (V_a) can be expressed as

$$V_a = \left(1 - \frac{1}{3} sin\theta\right) sin\theta \cdot \pi \left(\frac{r}{cos\theta}\right)^3 + \pi r^2 (H - h) \quad (3)$$

By combining equations (3) and (4), we can determine the height of SC structures as a function of compressor pressure (P_c)

$$h = \left(1 - \frac{P_0}{P_L + P_C}\right) H + r \cdot tan\theta \left(sec\theta - \frac{1}{3} tan\theta\right) \quad (4).$$

Adhesion Measurements. Using a custom-built equipment (Adhesion tester, Neo-Plus, Korea; see Figure S5a), adhesive patches (1×1 cm^2) with different geometries were attached with a preload of 3.5 N/cm^2 to the Si wafer and glass until failure occurred in the pull-off direction. To test our device's adhesive performance in different conditions, we measured the normal adhesion forces on the substrate in dry (relative humidity ~50 %) and underwater conditions (see the inset images in Figure S5b). Specifically, the external preload (F_p) applied to the contact between the sample and the substrate is assumed to a positive force as shown in Figure S5b. The preload (3.5 N/cm^2) was applied to the sample for 5 seconds, wherein the preload corresponds to gentle pressure exerted by human touch. After removing the preload, the negative force corresponding to the pull-off force F_{ad} was obtained by detaching the samples from the substrate. For the peel-off test, the μ-SCs adhesive patches (thickness: 150 μm; area: 2×3 cm^2) with different geometries were bonded to a rigid string and attached to the Si wafer as shown in Figure S6. Samples were lifted off at a constant test velocity of ~1 mm/sec with the custom-built equipment, and force-displacement profiles were measured. All measurements were repeated at least 10 times at ambient temperature, with average values plotted throughout all figures.

Detailed derivations for wet adhesion of the μ-SC-based adhesive. Total adhesion of the octopus-inspired adhesive with unfoldable 3D micro-tips can be calculated by the sum of suction stress (σ_s) and capillary interaction (σ_c): $\sigma_{total} = \sigma_s + \sigma_c$. Here, the van der Waals adhesion is negligible for underwater adhesion.[5] As the artificial adhesive is attached, the tailored 3D micro-tips could be unfolded to increase the effective contact-area on the surface, expressed with $\pi r'^2$(see Figure 2j(i) and Figure S8a). Here, the radius of the unfolded tip r' is achieved by the curvature of 3D micro-tips in μ-SCs (Figure S8a), given by

$$r' = \frac{\pi R}{2}\left(1 - \frac{\theta}{90}\right) \quad (8)$$

To prevent the interference among μ-SCs during their unfolding behaviours, we designed our adhesive patches ($r' - r < d$; see Figure S7-8). Thus, the μ-SCs cannot physically contact each other in all experiments when completely unfolded (Figure S7b). In case of capillary interaction (σ_c), the inside of suction chambers could widen and reach vacuum state during attachment, resulted from the deformation of elastic pillars by an applied preload (see Figure 2j(i) and Figure S7). Herein, adhesion via capillary-bridges (σ_c) between engaged parallel surfaces is calculated with the dynamic contact area ($A_{dynamic} = \pi(r'^2 - r_v^2)$) of enlarged tips, which may be expressed as[6]

$$\sigma_c = \pi(r'^2 - r_v^2)\gamma n \cdot \left(\frac{cos\theta_1 + cos\theta_2}{h\prime} + \frac{2}{r\prime - r_v}\right) \quad (9)$$

Here, γ is the surface tension of water (~0.072 J/m^2), n is the number of μ-SCs per unit area (~5×10^3 #/cm^2), θ_1 is the contact angle of D.I. water on the substrate (silicon wafer), θ_2 is the contact angle of D.I. water on the μ-SC surface (s-PUA; see Fig. S9), and h' is the height of the liquid film (~0.25 μm).

Next, the suction stress (σ_s) is defined as

$$\sigma_s = \Delta P_{max} \cdot A_v \cdot n. \quad (10)$$

As μ-SC structures are pulled off, void formation occurs inside the structures (see images of Figure 2j(ii) and Figure S7). Here, ΔP_{max} is the pressure difference between the ambient pressure and the pressure inside the μ-SC (nearly creating a vacuum state; ~101 kPa) and A_v is the area of the void inside of suction chamber (πr_v^2). Since total adhesion is sum of suction stress and capillary interaction, σ_{total} can be obtained with equation (9) and (10)

$$\sigma_{total} = \Delta P_{max}\pi r_v^2 n + \pi(r'^2 - r_v^2)\gamma n \cdot \left(\frac{cos\theta_1 + cos\theta_2}{h\prime} + \frac{2}{r\prime - r_v}\right). \quad (11)$$

In underwater condition the assistance of capillary-bridges between widened concave-tips of suckers and an engaged surface could be expected due to the existence of residues of water molecules, preventing air leakages.[7] In particular, the contact area between an unfolded 3D micro-tips and engaged surface, which is the dynamic contact area ($A_{dynamic} = \pi(r'^2 - r_v^2)$), would change as the adhesive is pulled off. The suction effect may be estimated by the void area ($A_v = \pi r_v^2$), whereas the capillary interaction (σ_c) may be expressed as a function of $A_{dynamic}$. By monitoring r_c, we can predict the suction stress (green line), capillarity adhesion (blue line), and total adhesion strength (red line) of the bioinspired adhesive with μ-SCs (n ~5×10^3 #/cm^2) as plotted in Figure 2k. This is well in agreement with our experimental data (black dots).

Preparation and characterization of pigskin. The postmortem pigskins for adhesion tests were obtained from a local slaughterhouse (Jidong, Korea) and stored frozen, as following well-established methods [7, 8]. Pig cadaver grafts cut in ~2 × 2 cm^2 area were immersed in DI water for 2 hours. DI water was then removed from the surface of pigskins with blotting paper to use as the substrate for measuring the adhesion of the PDMS-based SC-adhesive. Micro-scale roughness profile of the pigskin surface was determined using a cross-sectional scanning electron microscope (SEM) image. We collected the vertical distances (y_i) from the mean-line of surface step-height corresponding to each point of the horizontal displacements and calculated the root-mean-square (RMS) roughness using atomic force microscopy (AFM), given by $R_{RMS} = \sqrt{\frac{1}{n}\sum_{i=1}^{n} y_i^2}$ as shown in Figure 3c.

Measurement of adhesion forces with pigskin. With well-established methods,[7, 8] postmortem pigskins may be used for various adhesion tests (see Figure S12). This is because pigskin is known to have similar morphological features to human skin.[7] After preparing the pigskin through the above method, flat grafts of pigskin were affixed to all areas underlying the surfaces using cyanoacrylate glue for adhesion tests (see Figure S12). The μ-SC patch or non-patterned patch was glued onto the opposing jig of the mechanical tester. Using samples of the same size (1 cm x 1 cm), pull-off adhesion tests on pigskin were performed with the custom-built equipment (Adhesion tester, Neo-Plus, Korea) as shown in Figure S5. The test samples were applied to the surface of pigskin with preload 3.5 N/cm^2 for 5 seconds. The adhesion force corresponding to the pull-off force was then obtained by detaching the test samples from the dry or wet pigskin (Figure 3d and S11a). For the peel-off test, the patch with μ-SCs and non-patterned patch were bonded to a rigid string and attached to the epidermis of pigskins as shown in Figure S12b. Afterwards, samples were lifted off, as the displacement was controlled at a constant velocity of ~1 mm/sec with the custom-built equipment. The pull-off and peeling adhesions on pigskin were measured on both dry skin (relative humidity ~50 %) and wet pigskin covered with droplets (40% of areal fraction). All pigskin experiments were repeated at least 10 times by randomly selecting from more than 10 pieces of postmortem pigskin at ambient temperature, and average values were plotted. All animal experiments were carried out in accordance with the guidelines of the Animal Welfare Act and the Guide for the Care and Use of Laboratory Animals, following protocols approved by the Institutional Animal Care and Use Committee (SKKUIACUC-2016-10-0001-2) of Sungkyunkwan University.

Demonstration of clean attachment properties of skin patch on the skin. To investigate the clean attachment properties on skin, our adhesive of the 3cm × 3cm size was attached to the back of volunteers' hands for 6 hours with their agreements via the approved IRB. From then, we monitored skin surface to observe any chemical residues, traces of dust particles, and other impurities, after the adhesive was used and removed from the hand. The μ–SC patch showed almost no signs of contaminations after detachment. Following school board policies, we initially received an approval from the Institutional Review Board (IRB) for both subject and parental assent (IRB approval number: SKKU 2016-10-010-001).

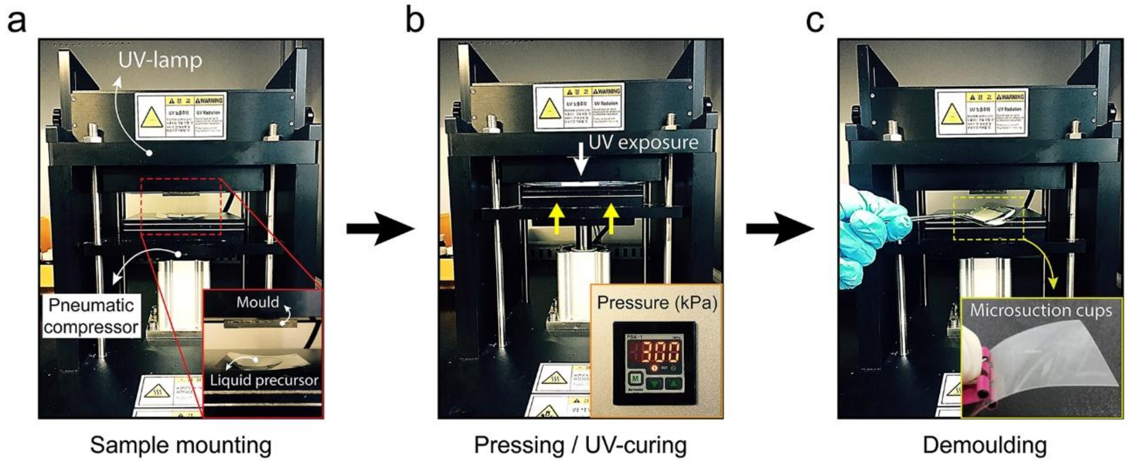

Figure S1. Photographs showing detailed fabrication process with a custom-built equipment connected to a pneumatic compressor. a) s-PUA liquid precursor was spread onto the PET film (thickness: 50 μm) after the prepared molds were fixed to the upper side in the pneumatic compressor. b) Pressing and curing a precursor-coated PET film (~300 kPa). c) Demolding the adhesive with μ-SCs.

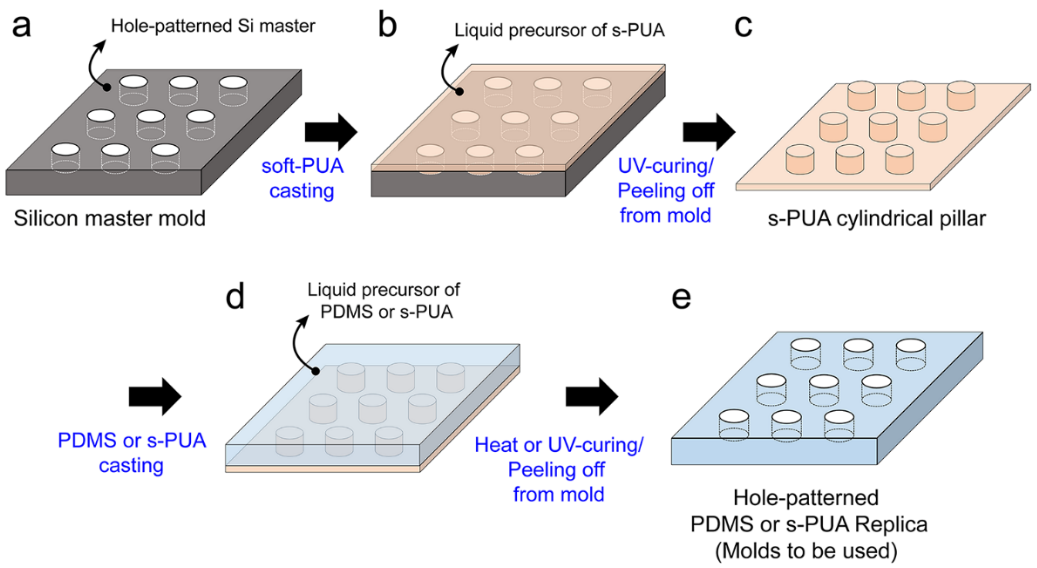

Figure S2. Schematic illustrations showing the preparation of various hole-patterned molds (PDMS and s-PUA) fabricated using a silicon master mold.

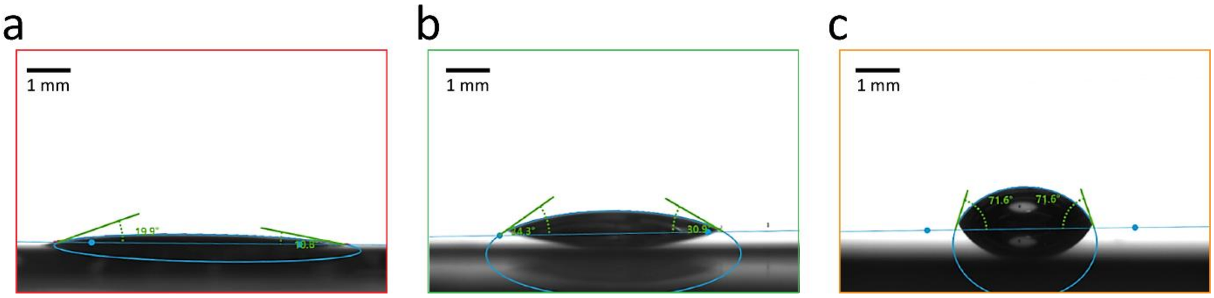

Figure S3. Optical images for contact angles of a 1 μL liquid droplet (s-PUA precursor; MINS-301RM) on different surfaces of a) s-PUA, b) FOTCS-treated silicon, and c) PDMS.

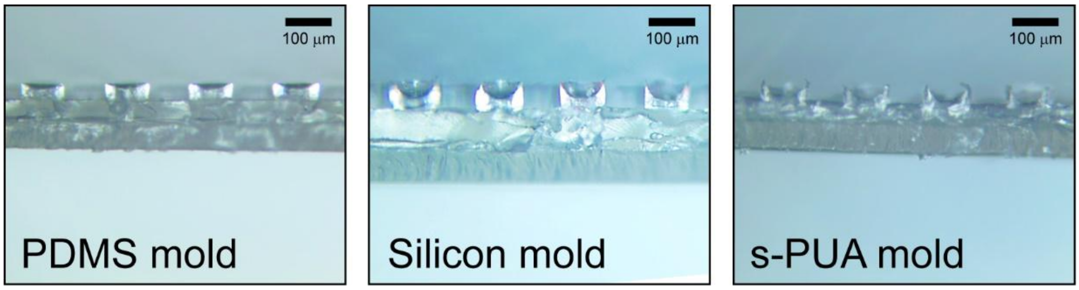

Figure S4. Cross-sectional images of various μ-SCs architectures (100 μm in diameter) based on PDMS, silicon, and s-PUA (from left to right).

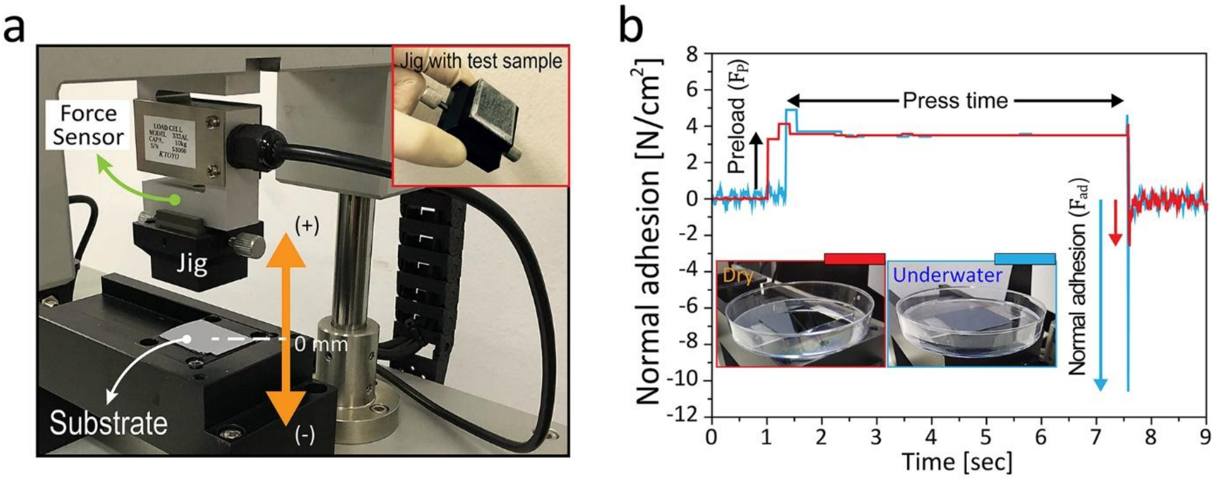

Figure S5. Measurements of pull-off adhesion strength. a) Pull-off adhesion test using custom-built equipment. The inset photograph shows an adhesive sample affixed to the jig. b) Representative time-dependent profiles of pull-off adhesion measured on dry and underwater surfaces. A uniform preload of 3.5 N/cm^2 is applied to the samples.

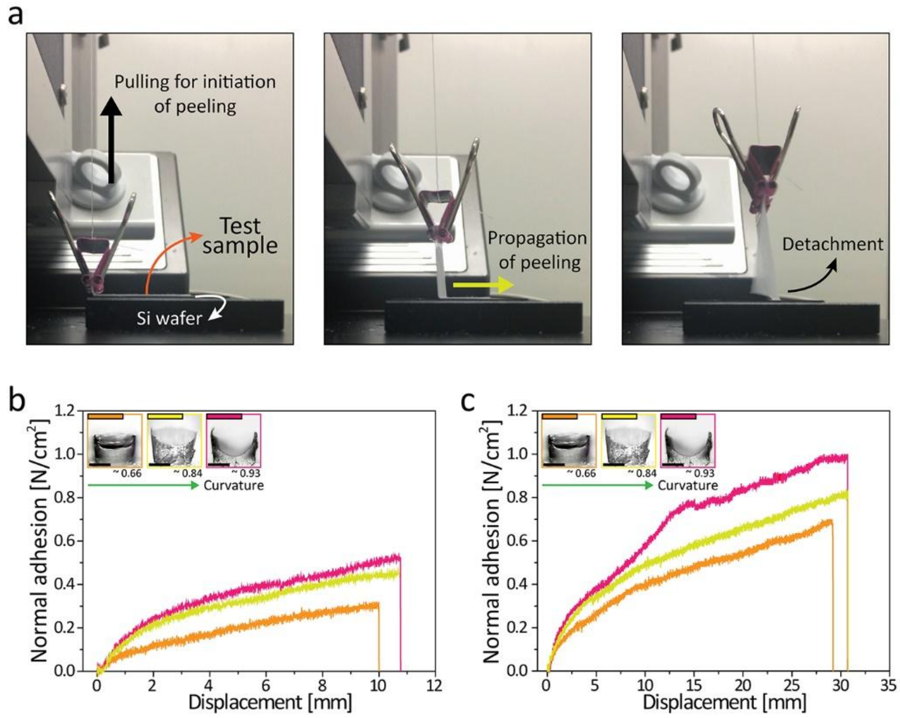

Figure S6. Measurements of peel-off adhesion strength. a) Peel-off adhesion test using custom-built equipment. b-c) Representative displacement-dependent profiles of peel-off adhesion in dry (b) and underwater (c) conditions.

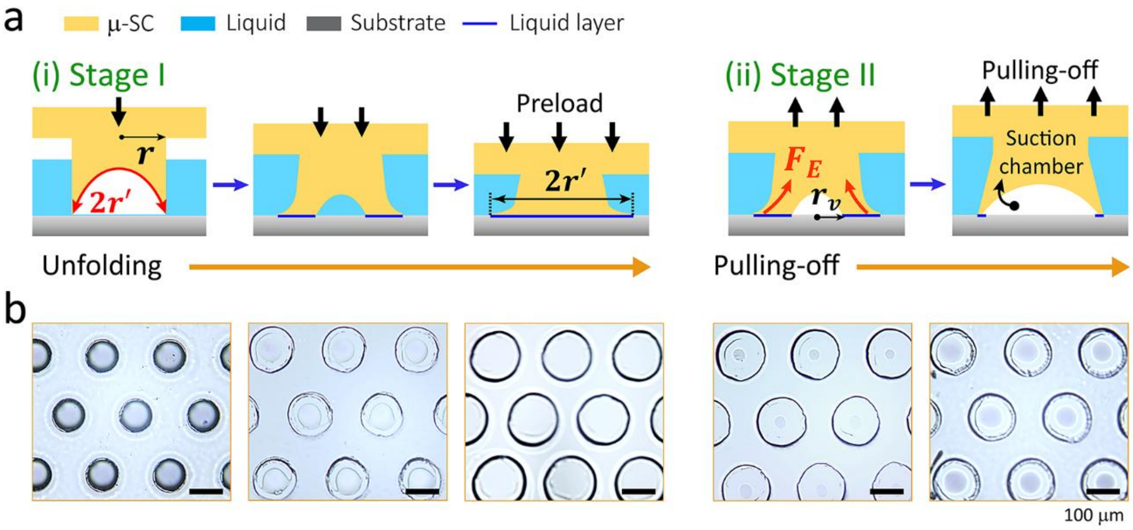

Figure S7. Detailed mechanisms underlying behavior of μ-SCs under wet conditions. a-b) Detailed unfolding and pulling-off processes a) of 3D micro-tips in a μ-SC and b) corresponding images of the array of μ-SCs on a wet surface.

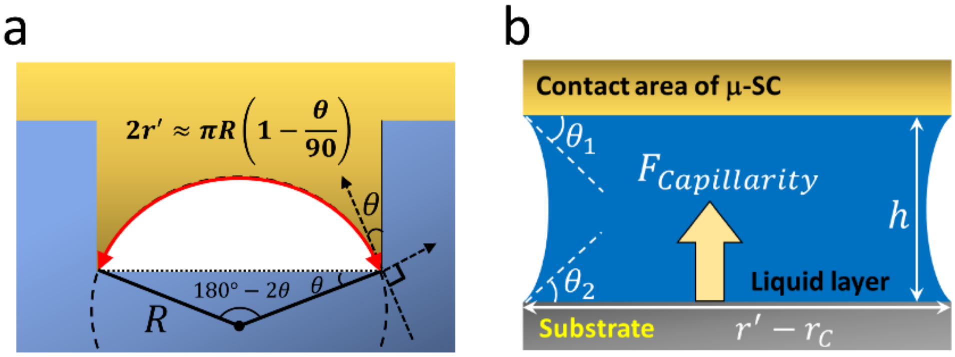

Figure S8. a) The illustration to estimate the curvature and r' of unfoldable 3D micro-tips. b) Schematics for capillary-assisted adhesion resulting from the liquid-bridge model between two parallel surfaces (blue line in Fig. 2j).

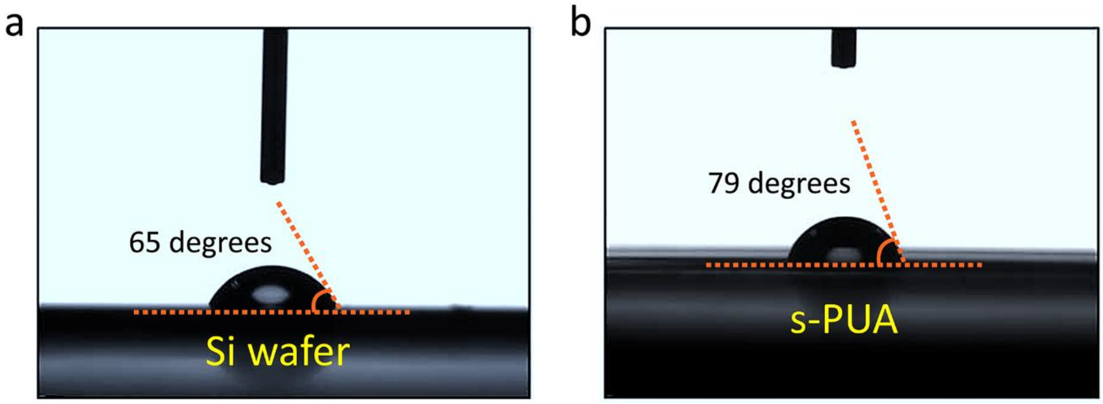

Figure S9. Measurements of contact angles of D.I. water droplet on a) silicon wafer and b) s-PUA surface.

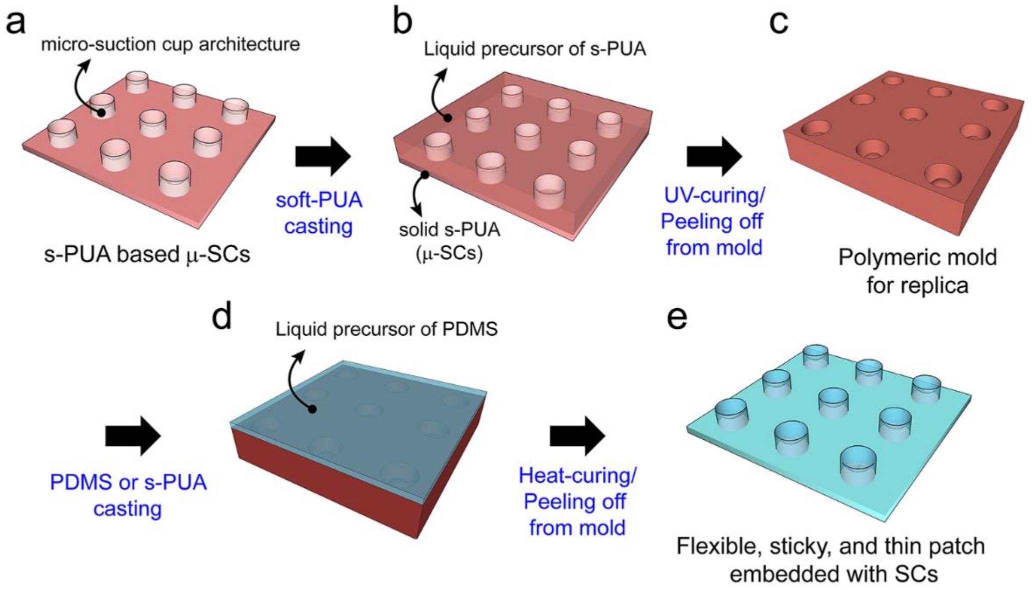

Figure S10. Schematic illustrations showing the detailed process for fabricating octopus-like biocompatible PDMS-based adhesive with enlargeable 3D micro-tips and μ-SCs (100 μm in diameter and 75 in μm height: 3 cm × 3 cm and ~600 μm thickness) by utilizing a simple replication technique.

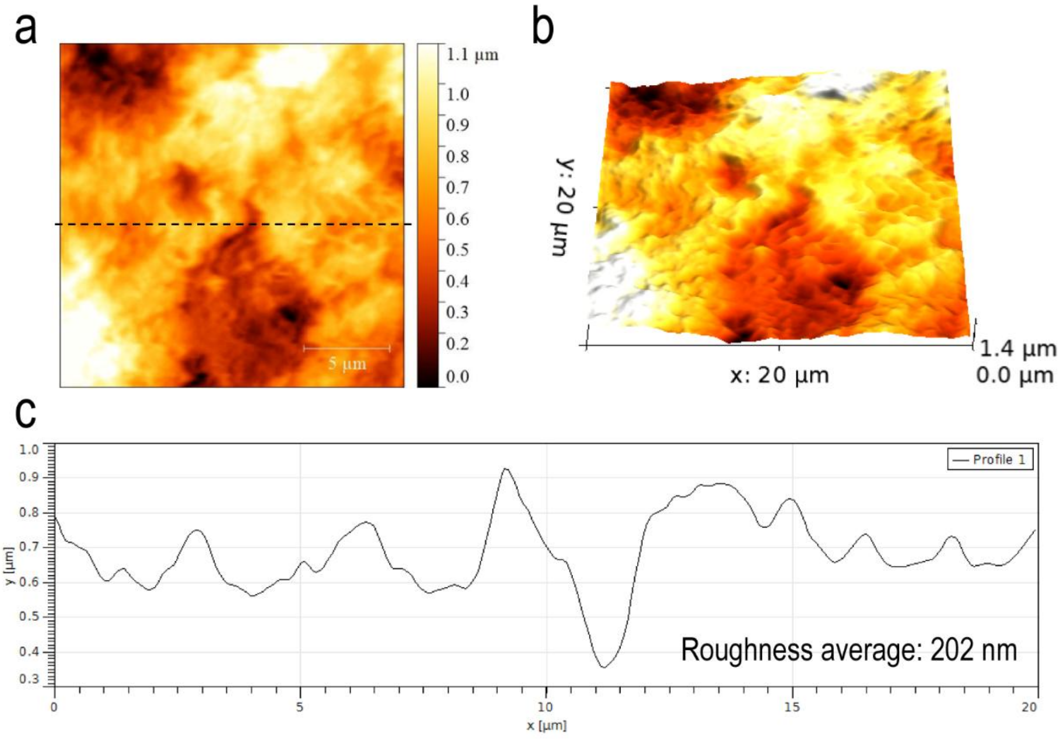

Figure S11. Characterization of the rough pigskin surface with hierarchical topography at sub-micro, or nano-scales. a) Two- and b) three-dimensional AFM images of a typical pigskin morphology with hierarchical roughness. c) Surface profile of the pigskin.

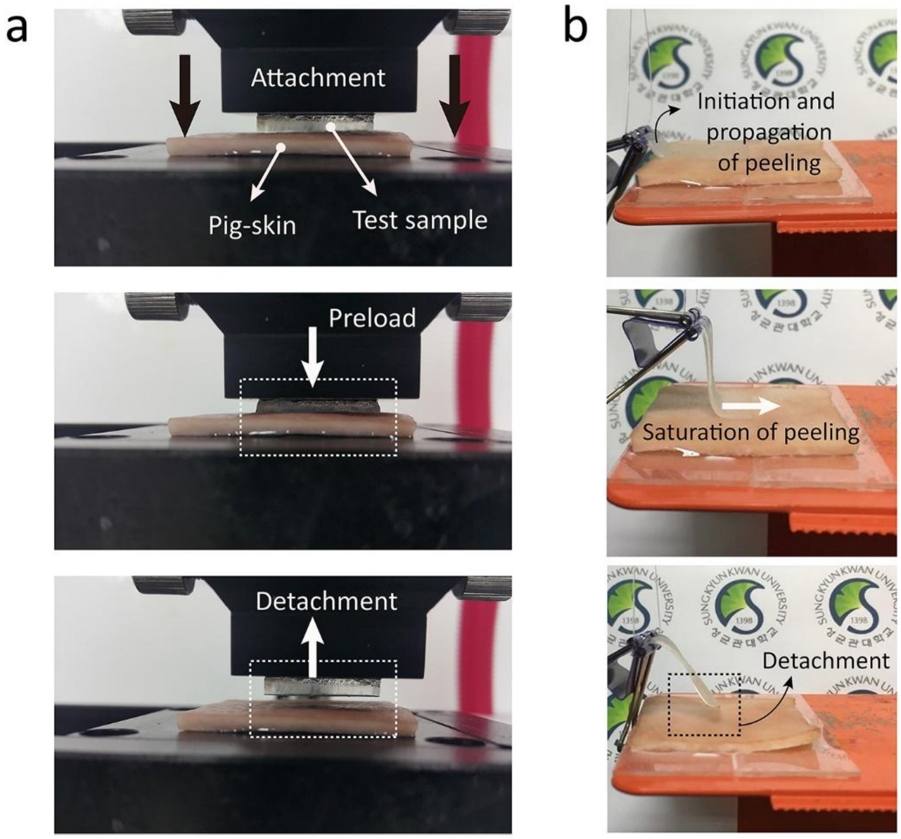

Figure S12. Detailed adhesion measurements in a) pull-, and b) peel-off directions. Photographs showing the octopus-inspired patch (100-μm diameter) on a pigskin.

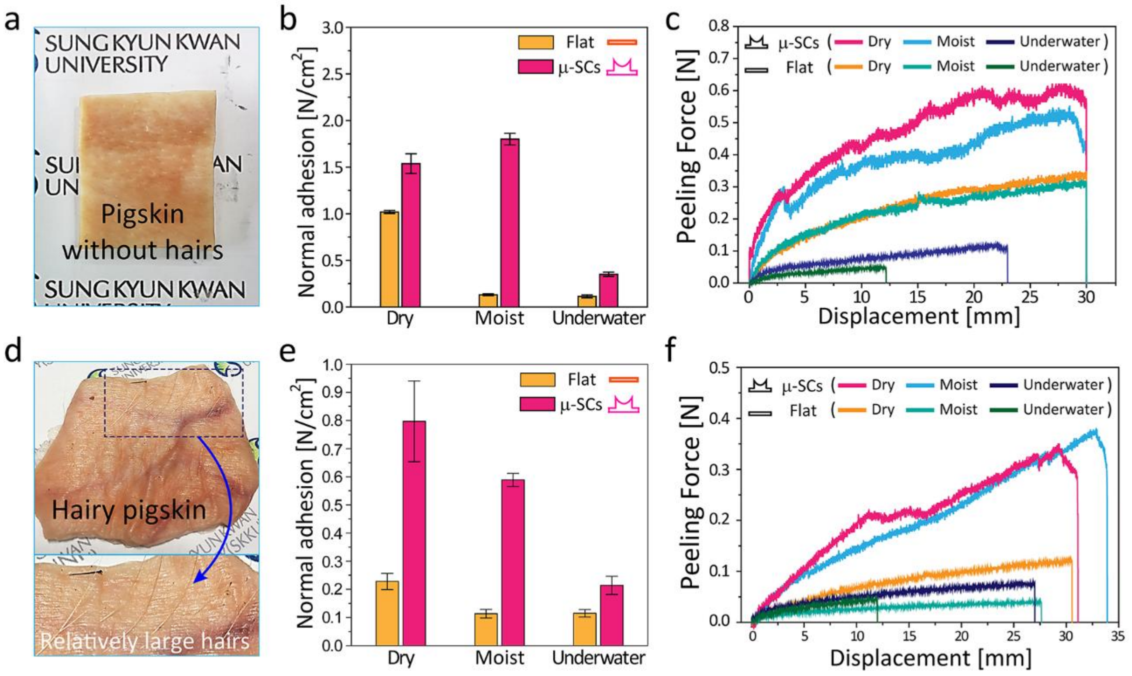

Figure S13. a) Photograph of pigskin. b) Pull-off strengths for a PDMS-based μ-SCs and a non-patterned PDMS-patch against a rough pigskin in dry, moist, and underwater conditions. c) Representative displacement-dependent profile of peeling adhesion on a hairless pigskin in dry, moist, and underwater conditions. d) Photograph of pigskin with relatively large hairs. e-f) Comparison of pull-off (e) and peel-off strengths (f) between a PDMS-based μ-SCs and a non-patterned PDMS-patch against a hairy, rough pigskin in dry, moist, and underwater conditions.

REFERENCE

[1] S.-J. Choi, P. J. Yoo, S. J. Baek, T. W. Kim, H. H. Lee, Journal of the American Chemical Society 2004, 126, 7744; P. J. Yoo, S.-J. Choi, J. H. Kim, D. Suh, S. J. Baek, T. W. Kim, H. H. Lee, Chemistry of materials 2004, 16, 5000.
[2] H. Yoon, T.-i. Kim, S. Choi, K. Y. Suh, M. J. Kim, H. H. Lee, Applied physics letters 2006, 88, 254104.
[3] H. Han, S. Baik, B. Xu, J. Seo, S. Lee, S. Shin, J. Lee, J. H. Koo, Y. Mei, C. Pang, Advanced Functional Materials 2017, 27; M. K. Kwak, H. E. Jeong, K. Y. Suh, Advanced Materials 2011, 23, 3949.
[4] J. J. Balowski, Y. Wang, N. L. Allbritton, Advanced Materials 2013, 25, 4107.
[5] A. E. Kovalev, M. Varenberg, S. N. Gorb, Soft Matter 2012, 8, 7560; M. Varenberg, S. Gorb, Journal of the Royal Society interface 2008, 5, 383.
[6] E. Cheung, M. Sitti, The Journal of Adhesion 2011, 87, 547; B. He, Z. Wang, M. Li, K. Wang, R. Shen, S. Hu, IEEE/Asme Transactions on Mechatronics 2014, 19, 312.
[7] A. M. Barbero, H. F. Frasch, Toxicology in Vitro 2009, 23, 1.
[8] S. Y. Yang, E. D. O'Cearbhaill, G. C. Sisk, K. M. Park, W. K. Cho, M. Villiger, B. E. Bouma, B. Pomahac, J. M. Karp, Nature communications 2013, 4, 1702.
